# The Roles of Endo-Lysosomes in Unconventional Protein Secretion

**DOI:** 10.3390/cells7110198

**Published:** 2018-11-03

**Authors:** Juhyung Lee, Yihong Ye

**Affiliations:** Laboratory of Molecular Biology, National Institute of Diabetes and Digestive and Kidney Diseases, National Institutes of Health, Bethesda, MD 20892, USA; Juhyung.lee@nih.gov

**Keywords:** protein translocation, misfolding-associated protein secretion (MAPS), unconventional protein secretion, endo-lysosome, intercellular transmission of proteins, protein quality control, neurodegenerative diseases, chaperone-mediated autophagy

## Abstract

Protein secretion in general depends on signal sequence (also named leader sequence), a hydrophobic segment located at or close to the NH2-terminus of a secretory or membrane protein. This sequence guides the entry of nascent polypeptides into the lumen or membranes of the endoplasmic reticulum (ER) for folding, assembly, and export. However, evidence accumulated in recent years has suggested the existence of a collection of unconventional protein secretion (UPS) mechanisms that are independent of the canonical vesicular trafficking route between the ER and the plasma membrane (PM). These UPS mechanisms export soluble proteins bearing no signal sequence. The list of UPS cargos is rapidly expanding, along with the implicated biological functions, but molecular mechanisms accountable for the secretion of leaderless proteins are still poorly defined. This review summarizes our current understanding of UPS mechanisms with an emphasis on the emerging role of endo-lysosomes in this process.

## 1. Introduction

The survival of individual cells relies on adaptation in response to environmental cues, which requires functional interplays between macromolecules secreted into cell exterior and their receptors on cell surface. The localization of secretory proteins is generally achieved by a leader sequence (or signal sequence), often at or close to the NH2-terminus of nascent polypeptides emerging from the ribosome [1]. The concept of the secretory pathway, which involves a collection of membrane-encircled organelles in constant exchange of materials (e.g., lipids and proteins) via vesicular trafficking, has been concretely established [2]. However, it has also become evident that many proteins lacking leader sequence can nevertheless exit the cell via active secretion, and the list of proteins undergoing this so called unconventional protein secretion (UPS) process has grown longer and longer in recent years [3]. 

The first experimentally confirmed UPS cargo is interleukin-1β (IL-1β) [4,5,6], a cytokine that plays critical roles in innate immunity [7]. Accumulated evidence suggests that IL-1β secretion can occur via several routes, most of which involve vesicular intermediates such as microvesicles shed from the plasma membrane (PM), exosomes derived from late endosomes called multivesicular bodies (MVBs) [8,9,10], or, as demonstrated more recently, via secretory autophagosomes [11]. The secretion of IL-1β evoked by inflammasome agonist nigericin was enhanced by starvation-induced autophagy, which depends on the small GTPase Rab8a and Golgi reassembly-stacking protein of 66 kDa (GRASP55) [12]. Additionally, several studies showed recently that IL-1β can be released into cell exterior by direct translocation across the PM through a pore formed by gasdermin D (GSDMD) in activated microphage that have been exposed to inflammasome activators [13,14]. Finally, since the activation of IL-1β secretion is often associated with cellular stress that activates apoptotic signaling, to what extent IL-1β may be passively released due to cell death under inflammation conditions in vivo remains to be determined. 

Fibroblast growth factors (FGFs) are a family of secreted pro-survival factors essential for animal development and tumorigenesis [15]. Although most FGFs are secreted via the endoplasmic reticulum (ER)-Golgi-mediated conventional secretory pathway, several members (FGF-1, FGF-2 and FGF-9) do not contain leader sequence [16]. In contrast to IL-1β whose secretion is stimulated by cellular stress, FGFs are constitutively secreted, particularly from cancerous cells with high levels of expression [17]. Later studies by the Nickel group has delineated the FGF-2 secretion mechanism: FGF-2 can insert itself into the PM as an oligomer and form a pore with a toroidal architecture [18,19]. Using an in vitro protein translocation assay, they found that the translocation of FGF-2 oligomers through the PM requires sequentially interaction of FGF-2 with PI(4,5)P2 at the inner leaflet and heparan sulfate proteoglycans at the outer leaflet of the PM [20], but protein unfolding is dispensable for this process [21]. Membrane insertion of FGF-2 oligomers is regulated by phosphorylation of tyrosine 81 by non-receptor tyrosine kinase Tec, which is associated with the inner leaflet of the PM through its pleckstrin homology (PH) domain [22]. Both gasdermin D-mediated IL-1β secretion and auto-pore-mediated FGF-2 secretion belong to type I UPS, as cargos translocate directly across the PM during secretion [3]. Other proteins that have been recently listed as type I UPS cargos include HIV1-Tat (HIV trans-activator of transcription), annexin A2 and FGF-1 [23,24,25,26]. Intriguingly, when the secretion of FGF-2 or IL-1β is forced to go through the ER/Golgi pathway (e.g., by appending a leader sequence to these cargos), secreted proteins are non-functional due to deleterious posttranslational modifications [27,28,29]. These findings suggested that UPS might be evolved to secrete proteins sensitive to posttranslational modifications in the ER. 

The small lipid binding protein AcbA is a more recently reported UPS cargo [30]. The secretion of AcbA is evolutionarily conserved and is essential for *Dictyostelium discoideum* development [30]. Unlike cargos of type I UPS, AcbA secretion is mediated by type III UPS, which generally requires a vesicular intermediate [31]. Using yeast as a model system, Malhotra’s group genetically defined genes involved in the secretion of a yeast Acb1 orthologue [32]. Their study using budding yeast as a system suggested a model in which Acb1 is packaged into autophagosomes in an ATG gene-dependent manner. Subsequently, Acb1-containing autophagosomes either directly fuse with the PM or mature into late endosomes/MVBs, followed by the release of Acb1 to the extracellular space. This process is stimulated by nutrient deprivation and requires the GRASP protein Grh1 (the yeast orthologue of mammalian GRASP65 and GRASP55) [30]. Upon starvation, Grh1 is assembled into a structure termed “compartment for unconventional protein secretion” or CUPS for short, which appears to be encased in a saccule that is stabilized by snf7, a subunit of the endosomal sorting complex required for transport III (ESCRT-III) [33]. In addition to snf7, CUPS also contains phosphatidylinositol 3 phosphate (PI3P) and the ESCRT protein Vps23 [34]. Morphologically, it does not overlap with the ER, Golgi or endosomes, but autophagic proteins Atg8 and Atg9 were found in this compartment even though they are not required for CUPS formation. These findings suggested that CUPS might be specialized for the biogenesis of secretory autophagosomes in yeast [34]. How CUPS recruits cargos and whether other UPS cargos beside Acb1 utilize CUPS as a secretory intermediate are currently unknown. 

Strikingly, studies from the Lee laboratory revealed an interesting twist in mammalian UPS: under certain stress conditions, cystic fibrosis transmembrane conductance regulator (CFTR), a multi-spanning membrane protein known to be transported via the classical ER-Golgi dependent pathway, could be delivered to cell surface via a type VI UPS mechanism that bypasses the Golgi complex [35]. Although the precise mechanism underlying this process is unclear, it required several regulators that were shown to be critical for AcbA secretion in yeast, including GRASP homologues and ESCRT components [35,36], suggesting that this two protein trafficking processes may share at least one common step. 

With the completion of the human genome sequencing project, it has become clear that many more secretory proteins fall into the category of UPS [37]. A family of proteins named galectin is such an example [38]. Proteins of the galectin family are β-galactosidase-specific lectins found in both intracellular and extracellular space. They have diverse functions ranging from cell adhesion, proliferation, differentiation and apoptosis, which are influenced at least in part by their localization [39]. Although the mechanism of galectin secretion is not fully defined, multiple lines of evidence suggested that galectins may be capable of using different pathways to exit the cell. For instance, one in vitro study showed that galectin-3 can interact with membrane lipids and spontaneously penetrate lipid bilayer of liposomes [40], raising the possibility of direct translocation across the PM. In support of this model, secretion of galectin-3 was dependent on self-oligomerization, which is mediated by an N-terminal domain [41]. However, other studies have suggested that galectin-3 might be secreted through an endosome-like vesicle intermediate [42].

The research on UPS has gathered steam in recent years, particularly as misfolded cytosolic proteins, including many neurotoxic polypeptides associated with neurodegenerative diseases, were found to undergo UPS under proteotoxic stress conditions [43,44,45]. Given the widely observed accumulation of neurotoxic proteins in extracellular milieu in animal models of neurodegenerative diseases and in patients, and that uptake of misfolded proteins such as Tau, α-Synuclein, and superoxide dismutase 1 (SOD1) has been suggested to contribute to the pathogenesis of neurodegenerative diseases [46], it is tempting to speculate that secretion through UPS, normally under tight regulation, may become deregulated and thus contribute to the pathological accumulation or transmission of misfolded proteins during disease progression [47]. This review summarizes our current understanding on the role of endo-lysosomes in this process.

## 2. Lysosome or Endosome as a Secretory Compartment?

Lysosomes are single-membrane encircled organelles discovered by Christian de Duve in 1955 [48]. For a long time, its primary roles were thought to be in degradation and recycling processes utilizing luminal pH-dependent hydrolase activities [49]. Around the time when the degradative role of lysosomes was being defined, Gorge Palade, another great cell biologist of the last century established the endoplasmic reticulum (ER) as the launching platform of the secretory pathway [50]. As his followers revealed the detailed framework of vesicular trafficking in eukaryotic cells, it has become evident that ER-originated vesicles can either target proteins to the PM for secretion or deliver lysosomal resident proteins to lysosomes. Once in lysosomes, it was assumed that these proteins have reached their final destination. However, Gilbert Vaes, while studying the mechanism of bone resorption, observed that several acid hydrolases of lysosomes could be released into the medium to catalyze bone absorption [51]. Excretion of lysosomal enzymes was later confirmed by numerous studies [52,53,54], but the nature and biological significance of these observations were unclear. Meanwhile, several studies on *Tetrahymena pyriformis*, a phagotrophic ciliated protozoan, revealed similar release of hydrolases into the medium. In 1972, Miklos Muller showed that the release of hydrolases from *T. pyriformis* was caused by active secretion. Importantly, he used sucrose gradient centrifugation to trace the origin of the secreted hydrolases to a special population of “lysosomes”, and thus for the first time linked ‘lysosomes’ to a secretory process [55]. Subsequently, studies on cells of the immune system showed that upon activation, cytotoxic T cells and natural killer cells secreted cytolytic proteins that had been stored in secretory granules [56,57,58,59]. Because these secretory granules bore hydrolytic enzymes and lysosomal membrane proteins, the evidence was taken to suggest the existence of “lysosomal secretion”. Noticeably, studies by Cockcroft and colleagues also confirmed secretion of lysosomal enzymes from azurophilic granules in neutrophils and HL60 cells, which turned out to be controlled by calcium. In addition, they used a semi-permeabilized cell system to demonstrate that this secretion pathway is regulated by a GTP-dependent step involving the ADP-ribosylation factor 1 (ARF1), phospholipase D and a phosphatidylinositol transfer protein (PITP) [60,61,62]. Meanwhile, Andrews and colleagues showed that “lysosomal secretion” is actually not restricted to specialized cell types [63,64]. Thus, by the early 2000s, the organelle once described as a “garbage disposal can” was believed to also serve a secretory function.

Despite the growing numbers of evidence that support a link between lysosomes and protein secretion, it remains to be established whether it is the degradative lysosomes that also serve a function in protein secretion, or as speculated by Muller and others that a population of “lysosomes” are specialized to perform a protein secretion function. One major issue is that many early studies were done before the term ‘endosome’ was coined, as it only became known later that the biogenesis of lysosomes involves progressive maturation of late endosomes [65]. Therefore, the distinction between late endosomes and lysosomes is quite blurry. Additionally, many protein markers used to define lysosomes in the above-mentioned studies were later found to be more promiscuously localized in the endo-lysosomal system. One striking example is the lysosome-associated membrane protein 1 (LAMP1), a glycoprotein previously thought to be exclusively present at lysosomes, but it is now found in a much broader domain that includes both lysosomes and late endosomes [66,67]. Late endosome can clearly participate in protein secretion as its fusion with the PM releases intraluminal vesicles (ILVs), which become one of the several types of extracellular vesicles that are collectively termed ‘exosome’. Thus, it is difficult to exclude the possibility that the previously assumed “lysosomal secretion” actually originates from late endosomes or a population of late endosomes. Consistent with this view, the cytoplasmic fatty acid binding protein 4 (FABP4) was recently added to the list of clients of “lysosomal secretion”, but the cargo was actually found within both endosomes and lysosomes prior to secretion [68]. Because of the difficulty in drawing a clear line between late endosomes and lysosomes, we propose the term “endo-lysosome” to describe the acidified UPS compartment with characteristics of lysosomes and late endosomes.

## 3. Protein Trafficking to Endo-Lysosomes

**Figure 1:**
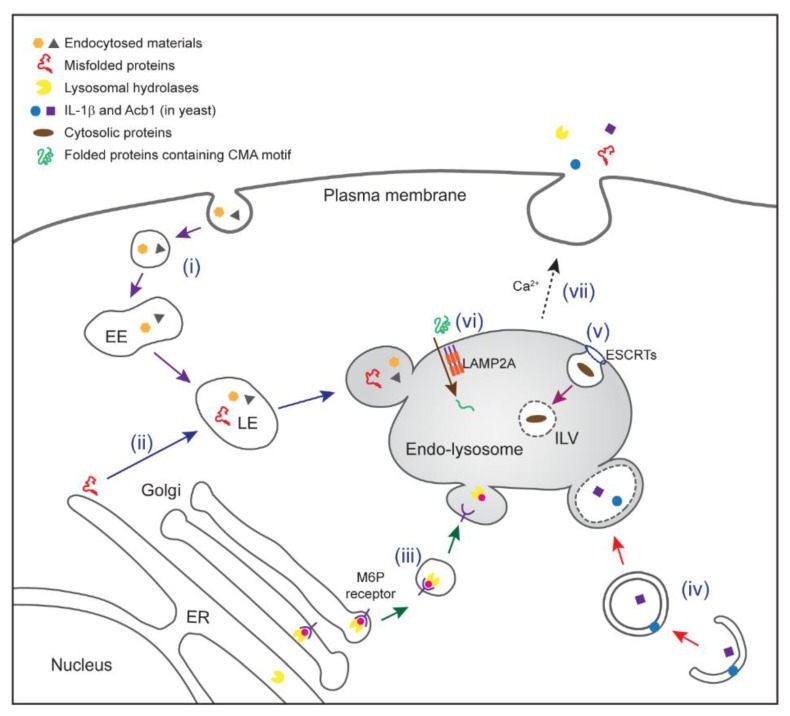
Multiple protein trafficking pathways to endo-lysosomes. (**i**) Endocytic trafficking. Fluid, solutes, macromolecules, plasma membrane components, and viruses can be internalized by invagination of the plasma membrane, forming vesicles. The vesicles fuse with each other, forming early endosomes (EEs) where cargo sorting occurs. Early endosomes move towards a perinuclear region and mature into late endosomes (LEs) as a result of continued membrane fusion, fission, and luminal acidification. A fraction of late endosomes bearing intraluminal vesicle (ILV) (endo-lysosome) may not have acquired a degradative function, and is rather specialized in protein secretion. Other late endosomes may fuse with lysosomes to degrade their contents. (**ii**) Misfolding-associated protein secretion (MAPS). Misfolded proteins are recruited to the endoplasmic reticulum (ER) surface by an ER-bound protein with a chaperone activity (e.g., Ubiquitin carboxyl-terminal hydrolase 19). Misfolded proteins are then transported into the lumen of endo-lysosomes for secretion. (**iii**) M6P receptor (MPR)-mediated delivery of lysosomal hydrolases. Most lysosomal enzymes undergo a mannose-6-phosphate (M6P) modification (pink circle) in the Golgi compartment. This allows them to bind to a MPR for sorting towards endo-lysosomes. In acidified endo-lysosomes, cargos are released from the receptor for delivery to lysosomes. Some of lysosomal hydrolases can be secreted to the extracellular space under specialized conditions probably via secretory endo-lysosomes. (**iv**) Secretory autophagy. Upon starvation, a compartment for unconventional protein secretion (CUPS; possibly equivalent to omegasomes in mammals) is formed near the ER exit sites to capture secretory cargos (e.g., IL-1β in mammal or Acb1 in yeast). GRASPs (Golgi reassembly and stacking proteins; Grh1 in yeast) and ATG proteins facilitate this process. Receptors that capture specific cargos have not been defined. Cargo-containing vesicles may fuse directly with the plasma membrane or first fuse with a secretory endo-lysosome prior to protein secretion. (**v**) Microautophagy (endosomal microautophagy in mammals). Cytosolic proteins are engulfed by invagination of endo-lysosomal membrane in either selective or non-selective manner. The initiation of microautophagy is mediated by several endosomal sorting complex required for transport (ESCRT) complexes. (**vi**) Chaperone-mediated autophagy (CMA). Heat shock cognate 71 kDa protein (HSC70) recognizes KFERQ-like motif in cytosolic proteins and targets them to lysosome-associated membrane protein 2A (LAMP2A). Multimerization of LAMP2A facilitates translocation of cargos in an unfolded form. CMA cargos are thought to be bound to lysosomal degradation. (**vii**) Endo-lysosomes or small vesicles derived from endo-lysosomes fuse with the plasma membrane in a Ca^2+^-dependent manner, which contributes to both conventional and unconventional protein secretion.

As endo-lysosomes stand at the crossroad between protein biosynthesis and triaging, its proteome is constantly modulated by multiple protein trafficking pathways (Figure 1). First, endo-lysosomes are the end-point of the endocytosis pathway, which takes up extracellular materials and downregulates cell surface molecules [69]. Endocytosis starts with invagination of cargo-bound PM by either clathrin-dependent or -independent mechanisms. Cargos enclosed in small vesicles are delivered to early endosomes where a triaging decision is made: while some cargos are recycled back to the PM through a recycling mechanism, the rest go through several endosome maturation steps, which are fueled by constant membrane fusion and fission events. As early endosome matures to become late endosome, its protein composition is constantly changed, and the internal pH is progressively decreased to ~pH 5, which is optimal for lysosomal hydrolases [70]. Late endosomes are thought to fuse with lysosomes, leading to the degradation of internalized cargos. 

Endo-lysosomes also receive proteins from the ER (Figure 1). The lumen of lysosomes harbors approximately 60 different hydrolases (refer to as acid hydrolases), which break down various biological substances including glycans, lipids, glycogen, and proteins. Most lysosomal enzymes are synthesized in the ER, and therefore need to be delivered to lysosomes via a mannose-6-phosphate (M6P) dependent ER-to-lysosome trafficking pathway [71]. M6P allows these enzymes to be recognized by M6P receptors (MPRs) in the trans-Golgi network (TGN), which recruit AP1/clathrin to facilitate the formation of small vesicles that carry lysosomal resident proteins. These vesicles then fuse with late endosomes, the acidic environment of which causes dissociation of M6PR from the cargos. While the receptors are recycled back to the TGN, lysosomal proteins continue their journal to reach lysosomes [72]. Interestingly, some proteins (e.g., β-glucocerebrosidase) may reach lysosomes via a M6P-independent mechanism [73,74]. 

Endo-lysosomes are also a destination for malfunctioned cytosolic contents to be eliminated by the cell. Cytoplasmic cargos such as unwanted polypeptides, protein aggregates, or damaged organelles can be internalized into lysosomes in eukaryotes through a highly conserved process known as autophagy [75] (Figure 1). Cells utilize autophagy to maintain protein homeostasis, especially under stress conditions such as amino acid starvation. The best characterized form of autophagy is macroautophagy, which is generally referred to as autophagy [76]. In this pathway, a portion of the cytoplasm is first sequestered within a double-membrane structure called phagophore, which expands to form autophagosome. This process is governed by more than 40 autophagy-related (ATG) proteins [76,77]. Autophagy can occur in either non-selective or selective manner. In the case of selective autophagy, cargos are recruited by autophagic receptors such as p62 (for ubiquitinated cargos), OPTN (Optineurin; for protein aggregates and bacteria), NIX (also known as BNIP3-like) and BNIP3 (BCL2 and adenovirus E1B 19-kDa-interacting protein 3) (for damaged mitochondria). These receptors interact with Atg8 (also named LC3 in mammals) to initiate phagophore formation and maturation. Matured autophagosome eventually fuses with late endosomes/lysosomes, resulting in the degradation of cargos sequestered within the inner membranes of the autophagosome. 

In addition to receiving cytoplasmic proteins from autophagosome, late endosomes can also directly take up cytoplasmic proteins when the membranes of late endosomes invaginate inwards, budding into the lumen to form intraluminal vesicles (ILVs) that sequester a portion of the cytoplasm (Figure 1). As a result, late endosomes become MVBs. This form of autophagy is termed microautophagy [78]. Genetic and biochemical studies in yeast have revealed multiple players involved in invagination, fission, or sealing of the membranes during MVB biogenesis [79]. Specifically, the ESCRT complexes cooperate with accessory proteins to recruit cargos and to pinch off the invaginated membranes from the limiting membranes. Microautophagy was initially thought to be non-selective, but a recent report showed that some cytosolic proteins can be delivered to late endosomes by HSC70, which may define a form of selective microautophagy [80]. 

Lastly, chaperone-mediated autophagy (CMA) is another form of autophagy that is capable of taking up cytoplasmic proteins. In contrast to membrane-mediated capture of cytoplasmic cargos in macroautophagy and microautophagy, clients of the CMA pathway translocate directly into the lumen of lysosomes via a protein translocation pore postulated to be formed by a single-spanning type I membrane protein named LAMP2A [81]. 

In short, late endosomes and lysosomes can receive proteins from both the secretory pathway and the cytoplasm. Therefore, once engaged in secretion, endo-lysosomes can inevitably contribute to both conventional and unconventional protein secretion. For cytoplasmic proteins to be secreted, depending on the mechanism by which they enter the lumen of endo-lysosomes (see below), cargos can be released into cell exterior either in small vesicles (for those encapsulated by MVBs) or in a vesicle-free form (for those translocated into the lumen of endo-lysosomes).

## 4. Protein Translocation across Endosome Membranes

**Figure 2:**
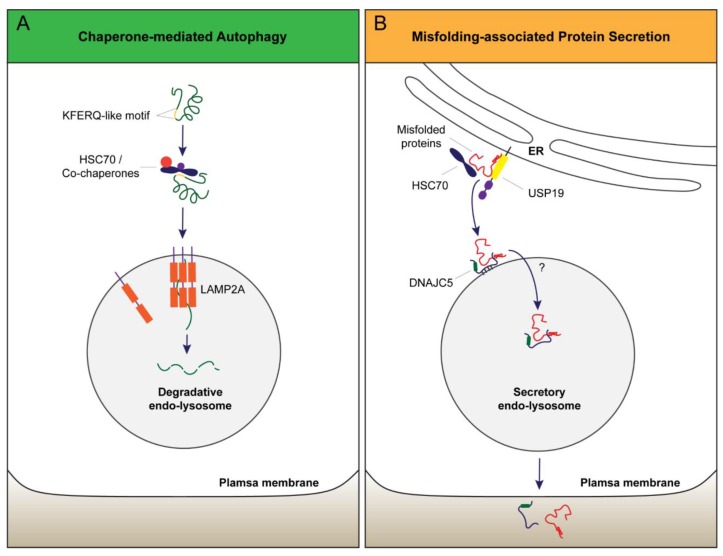
Models of protein transport into endo-lysosomes in CMA and MAPS. (**A**) CMA substrates bearing a KFERQ-like motif are recognized by cytosolic HSC70/co-chaperones. This complex is delivered to the lysosomal surface where the CMA receptor LAMP2A captures substrates via its cytoplasmic tail. Protein unfolding and LAMP2A multimerization are essential for the translocation of cargos into the lumen for lysosomal degradation. (**B**) In MAPS, an ER-associated protein with a chaperone activity (e.g., USP19) captures misfolded proteins. MAPS cargos are subsequently transported to the late endosomes by DNAJC5 (DnaJ heat shock protein family member C5), which associates with late endosome membranes via palmitoylation. DNAJC5 and the associated cargos are translocated into the lumen of vesicles by an unknown mechanism, but protein unfolding is not required for this process. Secretion through endo-lysosome releases DNAJC5 together with cargos into the extracellular space.

Ehrenreich and Cohn demonstrated in the late 1960s that in response to endocytosis of high concentrations of peptide, “lysosomes” within the cell increase dramatically in volume as a result of osmotic swelling, indicating that the lysosomal membranes are impermeable to macromolecules like peptides [82]. However, several reports in 1980s and 1990s showed that in mammalian cells certain cytosolic proteins can be imported into lysosomes for degradation, particularly when cells have undergone prolonged starvation or serum deprivation [83,84,85]. Thus, the membranes of ‘lysosomes’ must harbor a protein translocation machinery. Dice and Cuervo characterized stress-induced protein translocation in lysosomes using a combination of in vitro and cell-based assays. They showed that translocation of cytosolic proteins into lysosomes in this degradative pathway requires a penta peptide in cargo proteins and the HSC70 chaperone [86,87]. They therefore named this process chaperone-mediated autophagy (CMA) (Figure 2A). Their studies revealed a model in which HSC70 recognizes CMA cargos and targets them to the cytoplasmic domain of LAMP2A, which is localized on the surface of late endosome/lysosomes [88]. LAMP2A is encoded by one of the three spliced variants of the *LAMP2* gene. Although these variants are identical in most amino acid sequence (the only difference being in the transmembrane domain and the cytoplasmic tail), only LAMP2A, but not the other two variants are involved in CMA [88]. Once being targeted to LAMP2A, cargos are unfolded and translocated into the lumen of late endosomes/lysosomes. The mechanism of protein translocation is not fully understood, but it requires oligomerization of LAMP2A, which might be sufficient to form a protein translocation pore [88]. 

We recently reported misfolding-associated protein secretion (MAPS) that promotes protein homeostasis by eliminating misfolded cytosolic proteins when their production exceeds the rate of turnover by the proteasome [43]. In this process, misfolded proteins are first recognized by an ER-associated protein that has a chaperone activity (e.g., USP19). Misfolded cargos subsequently enter the lumen of late endosomes that are in close contact with the ER. This step is facilitated by HSC70 and DnaJ heat shock protein family member C5 (DNAJC5), with the latter being an endo-lysosome associated co-chaperone of HSC70 [45]. DNAJC5 then escorts cargos into the lumen of late endosomes for secretion to cell exterior [45] (Figure 2B). 

At first glance, the MAPS and CMA pathways appear similar as they both involve HSC70 and a protein translocation event at the membranes of late endosomes/lysosomes. However, several lines of evidence suggest that these are two distinctive pathways. The first distinction is in the mode of substrate recognition. The MAPS pathway shows a selectivity towards misfolded proteins as the initial substrate recognition involves an ER-associated chaperones, whereas the CMA pathway targets proteins bearing an exposed penta-peptide [89]. The CMA motif is clearly dispensable for MAPS: model substrates of the MAPS pathway such as a truncated GFP mutant or unassembled Ubl4A do not carry a CMA motif. Nevertheless, these proteins can be translocated into the lumen of late endosomes [43]; on the other hand, for Parkinson’s disease-associated protein α-Synuclein, although it has a CMA motif, this motif can be removed without affecting its secretion via the MAPS pathway [90]. Second, while HSC70 is necessary for targeting CMA substrates to lysosomes, HSC70 may serve as a co-chaperone for ER-associated USP19 to facilitate substrate recruitment to the ER membrane in MAPS since it interacts physically with USP19 on the ER surface [45]. Importantly, unlike CMA, which couples protein translocation to unfolding, protein translocation into late endosomes in MAPS does not require cargo unfolding because a chimeric protein containing α-Synuclein fused with DHFR is secreted regardless of the folding state of the DHFR domain [90]. Moreover, β-galactosidase fused to α-Synuclein can be secreted by MAPS in an enzymatically active form [90]. Lastly, in contrast to CMA activation in response to serum deprivation [84], MAPS is prohibited under serum starvation conditions [90]. Thus, it seems that there exist at least two distinct translocation pathways to import cytosolic proteins into endo-lysosomes.

## 5. Endo-Lysosome-Mediated Secretion and Human Diseases

Defects in lysosomal degradation are known to result in accumulation of protein aggregates in lysosomes, causing human diseases that are collectively termed lysosomal storage diseases. Since neither the mechanism nor the substrate scope of endo-lysosomal secretion is clear currently, whether genetic mutations that disrupt this process could cause human diseases in a similar manner remains to be determined. Nevertheless, studies on DNAJC5, a key MAPS modulator, have revealed a potential link between MAPS and ceroid lipofuscinosis [91,92], a neurodegenerative condition associated with accumulation of auto-fluorescent “lysosome-like materials” in neurons. Most disease-associated mutations are localized in a cysteine-rich segment that is essential for palmitoylation and thus endosomal association of DNAJC5. Since endosome localization of DNAJC5 is required for its function in MAPS [45], the MAPS pathway may serve a ‘regurgitation’ function to prevent build-up of cellular waste in the endo-lysosome system. This function might be particularly essential for neurons given that these terminally differentiated cells cannot use cell proliferation to dilute or rid of cellular wastes.

In addition to cell autonomous cytotoxicity, dysfunctions in endo-lysosomal secretion may also have non-autonomous effects on cell viability. Misfolded proteins released by MAPS, if not properly controlled, might contribute to neuroinflammation widely seen during neurodegeneration. In addition, MAPS may accelerate neurodegeneration by providing a means to spread protein conformation-associated cytotoxicity if misfolded proteins released by a stressed neuron bind to another cell and subsequently enter the recipient cell. 

Intercellular transmission of misfolded proteins has been wildly reported for neurodegenerative diseases, but the underlying mechanism has been obscure [93]. Active secretion of Parkinson’s disease-associated α-Synuclein was first noticed by Lee and colleagues. They showed that a small fraction of newly synthesized α-Synuclein is secreted from SH-SY5Y cells either in monomeric or aggregated forms via an unconventional protein secretion channel. They also noticed the presence of a small amount of α-Synuclein in intracellular vesicles, but whether these vesicles represent a secretory intermediate is unclear [94,95]. Secretion of α-Synuclein was later confirmed by several studies, but the underlying mechanism has been controversial. Emmanouilidou showed that a small fraction of secreted α-Synuclein was associated with exosome and concluded that the secretion of α-Synuclein might be coupled to MVB biogenesis [96]. However, the same study also showed that the majority of secreted α-Synuclein molecules were not bound to vesicles. Studies by us and others by contrast showed that the secretion of α-Synuclein is independent of exosome biogenesis [43,44]. Instead, cytosolic α-Synuclein can translocate into late endosomes, which serve as a secretory compartment to release α-Synuclein in a vesicle-free form [43]. 

Secretion of misfolded proteins associated with other neurodegenerative diseases have also been reported. In Alzheimer’s disease, Aβ peptide can be secreted from cells via exosomes [97] or secretory autophagy [98], whereas Tau was reported to follow either exosomes [99,100,101], PM-derived vesicles [102], or late endosomal secretory pathways [103]. It may even be secreted by direct translocation across the PM [104]. Mutant Huntingtin (mHTT) implicated in Huntington’s disease was shown to exit neuronal cells via a late endosomal/lysosome dependent unconventional secretory pathway. Interestingly, like cargos of the MAPS pathway, mutant HTT but not wild-type HTT was found in late endosomes/lysosomes [105], suggesting that mutant HTT may also be a MAPS substrate.

Albeit not formally confirmed in vivo, in ex-vivo studies, α-Synuclein released by MAPS can gain access to other cells via endocytosis and subsequently degraded by lysosomes in recipient cells [45]. Because under normal conditions, secretion via endo-lysosomes releases only a relatively small portion of the misfolded protein pool and because of endocytosis-mediated turnover, misfolded proteins secreted by UPS should have minimal impact on surrounding cells. However, as secretion through endo-lysosomes can be enhanced by many factors including lysosomal pH, cytoplasmic calcium concentrations, and stress such as proteasome dysfunction [10,106], the balance between the secretion of misfolded proteins and their elimination may be disrupted under stress conditions, leading to increased intercellular spreading of misfolded proteins in a prion-like manner. 

## 6. Conclusions and Perspectives 

As evidence for endo-lysosomes in UPS is mounting, more questions also arise. A key question is whether there exists a population of acidified endosomes that are specialized in protein secretion, and if so, what differentiates them from other endocytic vesicles or degradative lysosomes. Another central question is how cytosolic proteins are selected for secretion and how they are translocated into the lumen of late endosomes. Additionally, it remains a major challenge to reveal whether and how UPS may contribute to intercellular transmission of misfolded proteins in neurodegenerative diseases. Nevertheless, studies in this area will for sure be the most exciting frontier in UPS research, which may one day lead to a drug that benefit patients by halting or alleviating intercellular transmission of neurotoxic proteins.
